# Impaired Bicarbonate Transport via SLC26A3 and CFTR Downregulation Promotes Mucous Cap Formation in Sessile Serrated Lesions

**DOI:** 10.1007/s10620-025-09470-5

**Published:** 2025-10-17

**Authors:** Hiroyoshi Ota, Miharu Suzuki, Heiwa Tanabe, Yukiko Kusama, Ayako Seki, Etsuo Hara, Takeshi Uehara

**Affiliations:** 1 Department of Clinical Laboratory Sciences, Shinshu University School of Medicine, 3-1-1 Asahi, Matsumoto, Nagano 390-8621 Japan; 2 Department of Health and Medical Sciences, Shinshu University Graduate School of Medicine, 3-1-1 Asahi, Matsumoto, Nagano 390-8621 Japan; 3 Department of Pathology, Nagano Municipal Hospital, 1333-1 Tomitake, Nagano, 381-8551 Japan; 4 Department of Gastroenterology, Nagano Municipal Hospital, 1333-1 Tomitake, Nagano, 381-8551 Japan; 5 Department of Laboratory Medicine, Shinshu University School of Medicine, 3-1-1 Asahi, Matsumoto, Nagano 390-8621 Japan

**Keywords:** Bicarbonate transport, CFTR, Mucous cap, SLC26A3, Sessile serrated lesion

## Abstract

**Background:**

Sessile serrated lesions (SSLs) are recognized as precursors in the serrated neoplastic pathway leading to microsatellite instability-high colorectal cancer. A hallmark feature of SSLs on endoscopic examination is the mucous cap.

**Aims:**

We aimed to investigate the expression of the bicarbonate transporters SLC26A3 and CFTR in SSLs, using immunohistochemistry to elucidate their potential involvement in the pathogenesis of mucous cap formation.

**Methods:**

We analysed 14 SSLs from 12 patients using formalin-fixed, paraffin-embedded tissue sections. Histochemical staining with high-iron diamine-Alcian blue (HID-AB) and immunohistochemistry for MUC2, MUC5AC, SLC26A3, and CFTR were conducted.

**Results:**

In normal colonic mucosa, MUC2 was strongly expressed in goblet cells, whereas MUC5AC was absent. SLC26A3 was expressed on the apical membrane and in the cytoplasm of surface epithelial and upper crypt cells, while CFTR was localized to the apical membrane of epithelial cells along the crypt axis. In SSLs, crypts showed architectural distortion with mucin retention in the dilated lumina and an overlying mucous cap. HID-AB staining revealed the presence of sulfomucins and sialomucins. Goblet cells coexpressed MUC2 and MUC5AC, with MUC2 showing broader and stronger expression. These mucins showed a partially distinct and mutually exclusive distribution. Notably, SLC26A3 and CFTR expression levels were markedly reduced or absent in SSLs.

**Conclusion:**

SSLs showed a mixed acidic mucin phenotype and downregulated epithelial bicarbonate transporters. This may impair mucin expansion and hydration, leading to the formation of adhesive mucous caps, showing the potential link between defective bicarbonate transport and mucin physiology in SSLs.

## Introduction

Colorectal sessile serrated lesions (SSLs), previously known as sessile serrated adenomas/polyps, are major precursor lesions in the serrated neoplastic pathway that can lead to microsatellite instability-high colorectal carcinoma [1]. SSLs have been shown to present with a characteristic mucous cap during endoscopic examination—a feature observed in up to 60% of these lesions [2]. The mucous cap is one of the most common sentinel signs of SSLs. It is defined as a localized accumulation of mucus on the mucosal surface, which may be transparent, bile-stained, or contain cellular debris, and is removable by irrigation [2–4]. However, the mechanisms underlying the mucous cap formation remain unclear.

Recent studies have reported the critical role of epithelial ion transport in regulating the physicochemical properties of the intestinal mucus. Bicarbonate (HCO_3_^−^) is essential for mucin expansion, mucus hydration, and the formation of a properly structured mucus gel in the small intestine, which is rich in negatively charged sialomucins. Intracellularly, these anionic mucins are stored in a highly condensed state, stabilized by calcium (Ca^2+^) and hydrogen ions (H^+^), which shield the negative charges on the sialomucin molecules and form electrostatic cross-links. Upon secretion, HCO_3_^−^ facilitates of Ca^2+^ and H^+^ displacements, thereby unmasking the fixed negative charges on the sialomucin molecules and enabling rapid mucin unfolding, hydration, and gel formation [5]. In cystic fibrosis, defective cystic fibrosis transmembrane conductance regulator (CFTR)-mediated HCO_3_^−^ secretion disrupts this mechanism, resulting in the accumulation of thick, viscid, and adhesive mucus [6]. These observations highlight the physiological importance of HCO_3_^−^ secretion in maintaining a functional intestinal mucus barrier.

Given the essential role of HCO_3_^−^ in maintaining normal mucus properties, its regulated transport is particularly critical in the gastrointestinal tract, where mucosal protection relies on appropriate mucus consistency. In the intestine, solute carrier family 26 member A3 (SLC26A3), previously known as downregulated in adenoma (DRA), is the principal Cl^−^/HCO_3_^−^ exchanger, with its highest expression observed in the colon [7, 8]. Therefore, SLC26A3 is likely a key factor in intestinal mucin homeostasis.

With these considerations, we aimed to investigate the expression of CFTR and SLC26A3 in SSLs, using immunohistochemistry to explore their potential involvement in the pathogenesis of mucous cap formation.

## Materials and Methods

### Tissue Samples

Haematoxylin and eosin (H&E)-stained slides from consecutive cases of SSLs endoscopically resected at Nagano Municipal Hospital were reviewed. The slides and corresponding clinical data were retrieved from the archives of the Department of Pathology, Nagano Municipal Hospital. Among these cases, lesions exhibiting a mucous cap were identified, and only those with available formalin-fixed, paraffin-embedded (FFPE) tissue blocks were included in the analysis. A total of 14 lesions from 12 patients were analyzed. Histologically normal-appearing colonic mucosa from the same cases served as control colonic mucosa. The median patient age was 67.8 years (range, 36.7–79.2), with a male-to-female ratio of 8:4. Out of the 14 lesions, 10 were in the ascending colon, one in the transverse colon, and three in the descending colon.

### Histochemistry and Immunohistochemistry

All the specimens were fixed in 10% neutral-buffered formalin and embedded in paraffin blocks. Serial paraffin sections, each 3-μm thick, were prepared from each block. The sections were stained with H&E for histological examination and underwent histochemical and immunohistochemical staining.

To distinguish between sulfomucin-mucins bearing sulfate groups on their oligosaccharide side chains and sialomucin-mucins containing terminal sialic acid residues, paraffin sections were de-paraffinised, rehydrated, and stained using high-iron diamine–Alcian blue (HID-AB) at pH 2.5, as previously described [9].

The following primary antibodies were used for immunohistochemistry: MUC5AC (1:100, mouse monoclonal antibody, clone 45M1, Spring Bioscience, Pleasanton, CA, USA; Cat# E11080; RRID:AB_10978001 (same clone as Thermo Fisher MA5-12,178); validated according to Antibody Registry/Thermo Fisher datasheet), MUC2 (1:200, rabbit polyclonal antibody, sc-15334, Santa Cruz Biotechnology, Dallas, TX, USA; Cat# sc-15334; RRID:AB_2146667; validated at https://www.scbt.com/p/mucin-2-antibody-h-300), SLC26A3 (1:300, rabbit polyclonal antibody, HPA036055, SIGMA-ALDRICH, St. Louis MO, USA; Cat# HPA036055; RRID:AB_10602401; validated at https://www.sigmaaldrich.com/US/en/product/sigma/hpa036055), and CFTR (1:2000, mouse monoclonal antibody, clone L12B4, Thermo Fisher Scientific, Waltham, MA USA; Cat# MA5-11763; RRID: not available; validated at https://documents.thermofisher.com/TFS-Assets/LSG/certificate/Certificates-of-Analysis/MA511763_TF2585315B.PDF).

The paraffin sections were de-paraffinised, rehydrated, and treated with 0.3% hydrogen peroxide in methanol for 30 min at room temperature to block endogenous peroxidase activity. Antigen retrieval was conducted by heating the sections in Histofine antigen retrieval solution (pH 9.0; Nichirei Biosciences, Tokyo, Japan) in a Decloaking Chamber NxGen (Biocare Medical, Pacheco, CA, USA) at 110 °C for 10 min.

The slides were then incubated overnight at 4 °C with the primary antibodies. Subsequent incubation involved Novocastra Novolink™ (Leica Biosystems, Nussloch, Germany) for CFTR, and Histofine Simple Stain MAX PO Multi (Nichirei Biosciences) for the other antibodies. The visualisation was conducted using a 3,3ʹ-diaminobenzidine substrate solution. Finally, the sections were counterstained with haematoxylin, dehydrated, and mounted. The positive control tissues included normal gastric mucosa for MUC5AC, colon mucosa for MUC2 and SLC26A3, and pancreatic tissue for CFTR. Whole slide scanning and image acquisition were performed using the Leica Aperio AT2 whole-slide scanner and software (Leica Biosystems, Nussloc, Germany), equipped with a 20× Plan Apo objective lens (NA 0.75). Photomicrographs were captured with NDP.view2 Plus image viewing software (Hamamatsu Photonics, Shizuoka, Japan).

## Results

### Normal Colonic Mucosa

**Fig. 1:**
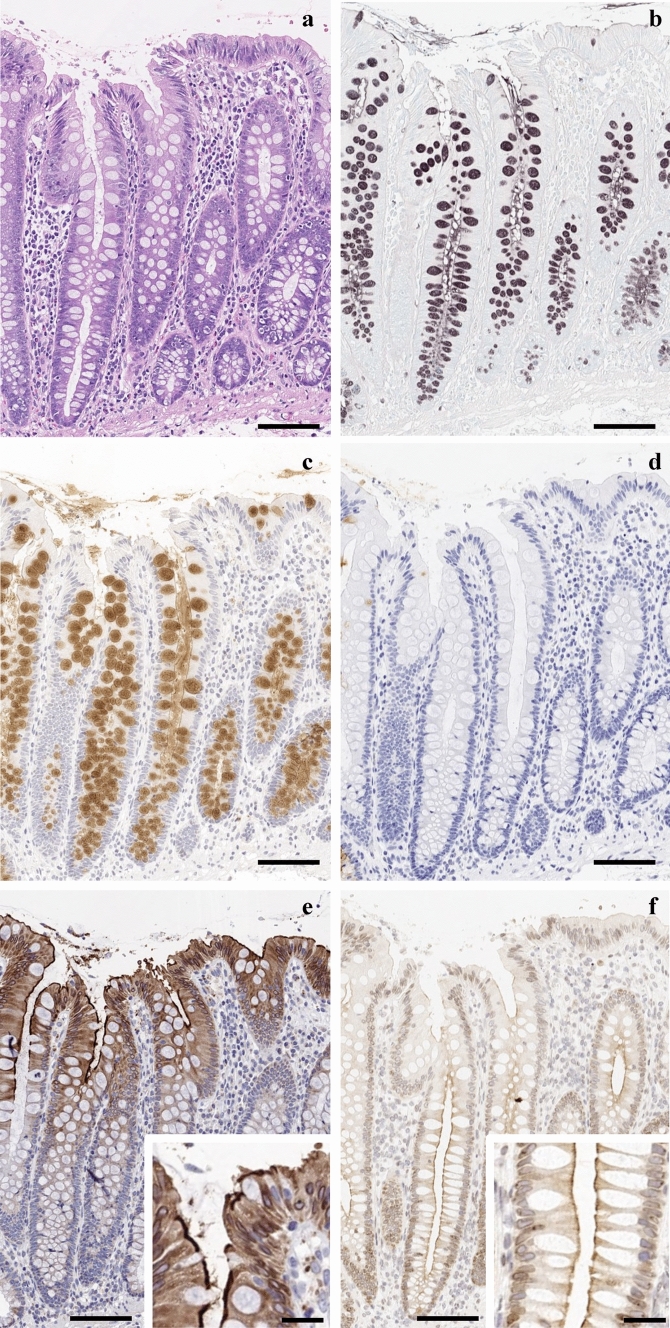
Histological and immunohistochemical features of normal colonic mucosa. Representative images of normal ascending colonic mucosa highlighting the distribution of mucin subtypes and epithelial ion transporters. **a** Haematoxylin and eosin staining shows well-preserved crypt architecture. The surface mucous gel layer is minimally retained. **b** High-iron diamine–Alcian blue staining shows goblet cells producing predominantly sulfomucins (brown to black) rather than sialomucins (blue). **c** Immunohistochemistry for MUC2 shows strong and diffuse staining in goblet cells throughout the crypts. **d** Conversely, MUC5AC, a marker of gastric-type mucin, is not expressed in normal colonic epithelium. **e** Immunohistochemistry for solute carrier family 26 member A3 shows strong expression along the apical membrane and within the cytoplasm of surface epithelial cells and those located in the upper third of the crypts. Inset: High-power view of the surface epithelium and upper third of the crypt. **f** Cystic fibrosis transmembrane conductance regulator immunoreactivity is restricted to the apical membrane of enterocytes in the crypts, with no detectable expression in the surface epithelium. Inset: High-power view of the crypt. Scale bar = 100 μm; inset: scale bar = 50 μm

The surface mucous gel layer covering the normal colonic mucosa was poorly preserved (Fig. 1a). Using HID-AB staining, sulfomucins were visualised in a spectrum ranging from brown to black, while sialomucins were stained blue. Goblet cells showed variable staining patterns depending on the crypt depth and colonic segment. Regional variations in sulfomucin expression were observed, with greater expression in the right colon than in the left colon (Fig. 1b).

Goblet cells showed immunoreactivity exclusively for MUC2, a marker of intestinal-type mucin (Fig. 1c). Conversely, MUC5AC, which is typically associated with the gastric foveolar epithelium, was not expressed in the goblet cells (Fig. 1d).

SLC26A3 showed strong apical membrane staining, with comparable levels of cytoplasmic staining in normal colonic mucosa. Its expression was localized to the surface epithelium and epithelial cells within the upper third of the crypts (Fig. 1e). In contrast, CFTR immunoreactivity was confined to the apical membrane of the epithelial cells within the crypts (Fig. 1f), and CFTR expression is not observed in the surface epithelium.

### SSLs

**Fig. 2:**
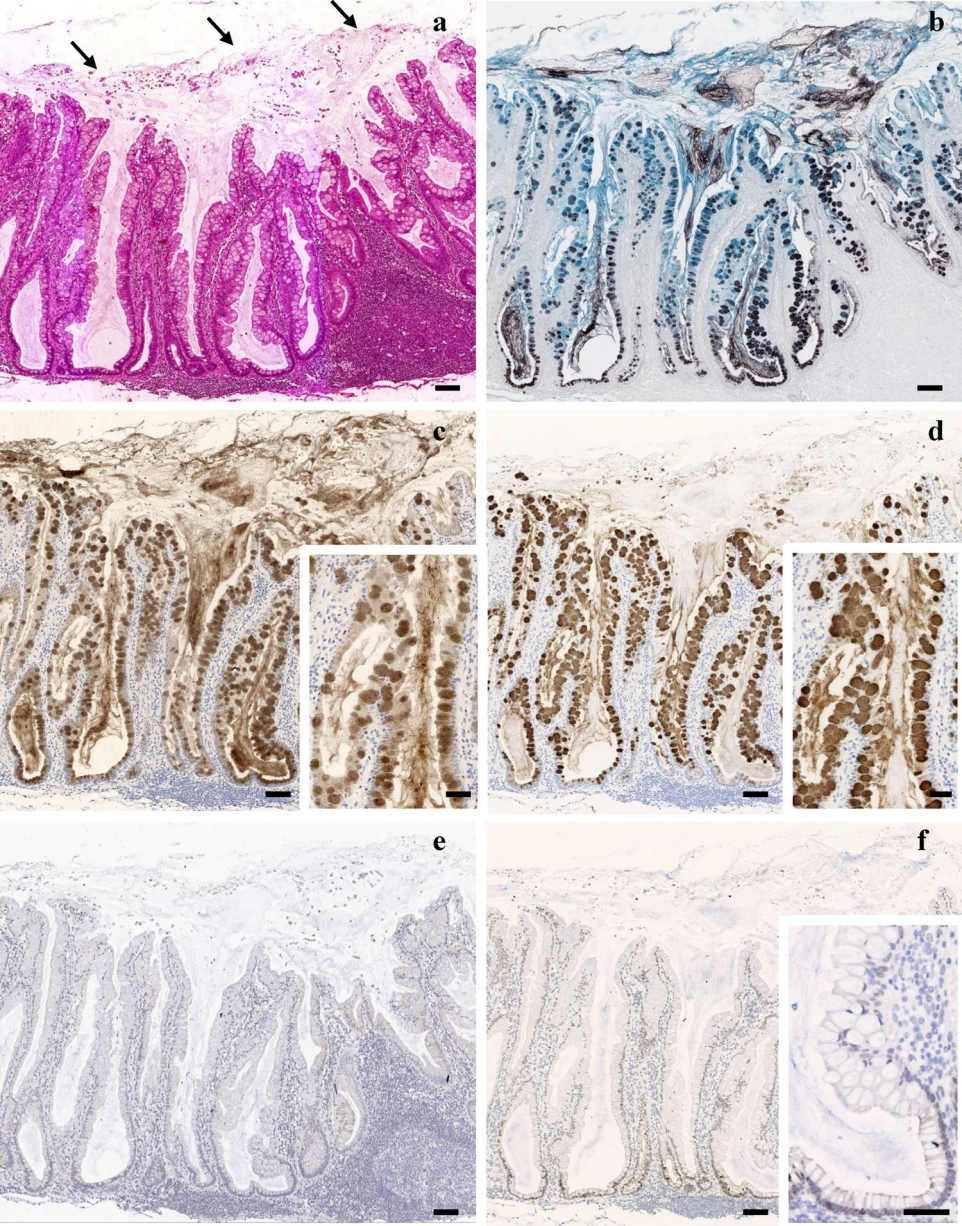
Histological and immunohistochemical features of sessile serrated lesions (SSLs). Representative images of SSLs showing characteristic architectural distortion and mucin-related alterations. **a** Haematoxylin and eosin staining reveals crypt dilation and irregular shapes, including “boot-shaped” and horizontally oriented crypts. Amorphous eosinophilic material is present within the dilated crypt lumina and on the luminal surface, corresponding to the mucous cap (arrows). **b** High-iron diamine–Alcian blue staining shows that this extracellular mucinous material contains sulfomucins (brown to black) and sialomucins (blue). **c**, **d** Immunohistochemical staining for MUC2 (**c**) and MUC5AC (**d**) shows co-expression in goblet cells within the lesion. Both mucin core proteins are also detected in the mucin pools within the dilated crypts and mucous cap, with MUC2 showing more extensive and intense staining, indicative of a predominance of intestinal-type mucin. MUC2 and MUC5AC display spatially distinct and partially mutually exclusive distribution patterns. Insets: High-power view of the crypts. **e**, **f** Immunohistochemistry for the epithelial ion transporters solute carrier family 26 member A3 (**e**) and cystic fibrosis transmembrane conductance regulator (**f**) reveals markedly reduced or absent expression in the lesional epithelium. Inset: High-power view of the crypts. Scale bar = 100 μm; inset: scale bar = 50 μm

The SSLs revealed a characteristic architecture, including dilated and irregularly shaped crypts, some of which show a "boot-shaped" or horizontally arranged configuration (Fig. 2a). Amorphous eosinophilic material was retained within the dilated crypt lumen and covered the luminal surface of the lesion, consistent with a mucous cap (Fig. 2a). HID-AB staining showed that goblet cells and this extracellular material contained sulfomucins and sialomucins in all SSLs, regardless of their location in the right or left colon (Fig. 2b). Sulfomucin-containing goblet cells were distributed in the middle and lower zones of the crypts (Fig. 2b).

Immunohistochemical analyses showed that goblet cells within the SSLs coexpressed MUC2 (Fig. 2c) and MUC5AC (Fig. 2d), indicating a mixed mucin phenotype. MUC2 (Fig. 2c) and MUC5AC (Fig. 2d) were detected in the mucin pools within the dilated crypts and in the mucous cap on the luminal surface. MUC2 (Fig. 2c) expression was more extensive and intense than that of MUC5AC (Fig. 2d), suggesting that colonic-type mucins predominate among the secreted mucins. Notably, the two mucins showed spatially distinct and partially mutually exclusive distributions (insets of Fig. 2c, d). Furthermore, immunohistochemical analysis showed that the expression of the epithelial ion transporters SLC26A3 (Fig. 2e) and CFTR (Fig. 2f) was markedly reduced or absent in the lesional epithelium.

## Discussion

In this study, we demonstrated that the mucins secreted within the crypts and mucous caps of SSLs are rich in acidic mucins, including sulfomucins and sialomucins, and contain MUC2 and MUC5AC. Notably, SSLs showed a reduced or absent expression of SLC26A3 and CFTR, which are key transporters responsible for epithelial HCO_3_^−^ secretion. This alteration may lead to abnormal mucin properties and contribute to the formation of the mucous cap.

Histological sections prepared from FFPE tissue typically lack the mucus gel layer that normally covers the surface of the non-neoplastic gastrointestinal mucosa, likely because of its high solubility and low adhesive properties [10, 11]. Conversely, an adherent mucus layer corresponding to the mucous cap remains visible on the luminal surface of SSLs even after FFPE processing. This difference implies that the mucus secreted by SSLs possesses enhanced viscosity and adhesiveness, enabling it to resist removal during standard histological preparations. These findings show the fundamental biochemical or structural differences in the mucus produced by SSLs compared with that of the normal colonic epithelium.

Consistent with previous studies [11, 12, 13] and further substantiated by our findings, colonic mucins are characterized by a high abundance of acidic mucins, which are primarily composed of sulfomucins and sialomucins. These glycoconjugates carry net negative charges under physiological conditions, owing to the presence of sulfate groups and sialic acid residues on their oligosaccharide side chains [14]. Such anionic properties are thought to significantly influence the physicochemical characteristics of the adherent mucus layer, especially its hydration dynamics, viscoelastic behavior, and capacity for spatial expansion along the colonic epithelium. These effects have been well-characterized in the small intestinal mucosa [5]; however, their precise roles in the colonic environment remain unclear.

Garcia et al. [5] demonstrated using an ex vivo mouse small intestine model that CFTR-mediated HCO_3_^−^ secretion is essential for mucin expansion, hydration, and the formation of a functional mucus gel. In the small intestine, which is enriched in negatively charged sialomucins, mucins are stored in a compacted intracellular state stabilized by Ca^2+^ and H^+^, which shield their polyanionic charges. Upon exocytosis, HCO_3_^−^ promotes mucin expansion and solubilization by chelating Ca^2+^ and neutralizing H^+^. This process exposes the fixed negative charges and allows rapid mucin unfolding and gel formation—steps essential for establishing an effective mucus barrier. These findings provide mechanistic insight into how defective HCO_3_^−^ secretion, as observed in cystic fibrosis, can lead to impaired mucus clearance and increased viscosity [5, 6].

Bicarbonate secretion is a physiological function of the colonic mucosa, primarily mediated by SLC26A3 and CFTR, with a predominant contribution from the former [8]. In this study, SLC26A3 and CFTR were expressed in the normal colonic epithelium, and their localization aligns with previous reports in human tissues [15–17]. In contrast, SSLs showed reduced or no expression of SLC26A3 and CFTR. This observation aligns with previous findings that have reported diminished or undetectable SLC26A3 mRNA expression in SSLs [18]. Given the critical role of these transporters in epithelial HCO_3_^−^ secretion, their downregulation may lead to impaired local HCO_3_^−^ availability. Consequently, the reduced capacity for mucin expansion and hydration may lead to the accumulation of highly viscous and adhesive acidic mucins, especially sulfomucins and sialomucins, on luminal surfaces. This pathophysiological alteration likely contributes to the formation of an adherent mucous cap, a hallmark feature of SSLs observed in endoscopic and histological settings.

Immunohistochemical analysis of serial sections showed that goblet cells within SSLs coexpressed MUC2 and MUC5AC, indicating a mixed mucin phenotype. Additionally, intraluminal mucin was retained within the dilated crypts, and the extracellular mucin forming the surface mucous cap stained positively for both markers. Notably, MUC2 immunoreactivity was more extensive and intense than that of MUC5AC, suggesting a predominance of colonic-type mucin. Furthermore, MUC2 and MUC5AC distributions appeared to be spatially distinct and partially mutually exclusive within the mucin pools, implying differential secretion and/or compartmentalization of these mucin subtypes.

In SSLs, MUC5AC and trefoil factor family 1 (TFF1) expressions have been reported [19, 20]. TFF1 is co-secreted with MUC5AC from gastric surface mucous cells. It possesses lectin-like properties and specifically binds to MUC5AC [21], but not to MUC6, which is secreted from gastric glands (cardiac gland, mucous neck, and pyloric gland cells) or colonic mucins [22]. Therefore, it is conceivable that in SSLs, secreted MUC5AC may be organized into a distinct structure through TFF1-mediated polymerization, thereby remaining spatially segregated from MUC2. Supporting this hypothesis, a similar mechanism has been described in the gastric mucus gel layer, where TFF2, which is co-secreted with MUC6 specifically binds to MUC6 but not to MUC5AC and is essential in forming a dense MUC6 network by remaining attached even after secretion [23]. This interaction is thought to contribute to the layered architecture of gastric mucus by preventing the homogeneous mixing of MUC5AC and MUC6 [10, 23, 24].

A limitation of this study is that FFPE tissue was used, which typically results in the loss of the surface mucus gel layer of the normal colonic mucosa during processing [11]. Therefore, a direct comparison between the native mucous layer of the normal colonic mucosa and the residual mucous caps observed in SSLs could not be conducted. Furthermore, portions of the mucous cap were likely lost during processing, potentially leading to an underestimation of results. To better preserve the mucus gel layer, fixation with Carnoy’s solution [10, 11] or formalin/picric acid solution [24] is recommended, with the latter being particularly compatible with subsequent immunohistochemical analyses. Prospective studies using these optimized fixation methods are necessary to elucidate the structural and functional properties of mucus gel layers in normal and neoplastic colorectal tissues.

In summary, we demonstrated that mucins secreted in SSLs, particularly those comprising the overlying mucous cap, are enriched in acidic intestinal-type mucins, including sulfomucins and sialomucins. These findings, alongside the observed loss of SLC26A3 and CFTR expression, support a mechanistic link between impaired epithelial bicarbonate transport, altered mucin composition, and mucous cap development. Further investigations using optimized fixation methods for mucin preservation are essential to fully elucidate the structural characteristics and clinical significance of mucous caps in serrated colorectal lesions.

## Data Availability

All data generated and analyzed during this study are available from the corresponding author upon reasonable request.
